# Cannabinoid CB1 receptor and mu-opioid receptor interaction: new insights from conditional knockout mice

**DOI:** 10.1038/s41386-025-02245-6

**Published:** 2025-09-25

**Authors:** Hannah Alton, Emily Linz, Guo-Hua Bi, Omar Soler-Cedeno, Maia Maras, Zheng-Xiong Xi

**Affiliations:** Addiction Biology Unit, Molecular Targets and Medications and Discovery Branch, National Institute on Drug Abuse Intramural Research Program, Baltimore, MD USA

**Keywords:** Chronic pain, Behavioural genetics

## Abstract

Co-administration of cannabinoids and opioids has been shown to enhance analgesic effects. However, the underlying mechanisms remain unclear. One hypothesis suggests that cannabinoid CB1 receptors (CB1R) and mu-opioid receptors (MOR) interact at the cell membrane or intracellular signaling level. This study aimed to test this hypothesis by examining CB1R-MOR colocalization and assessing whether the deletion of one receptor affects the other’s response to its ligand. Results from RNAscope in situ hybridization revealed that CB1R and MOR mRNAs exhibit distinct regional distributions in the mouse brain. Colocalization of CB1R and MOR was primarily observed in the paraventricular nucleus of the thalamus (PVT), where ~50% of vesicular glutamate transporter 2 (Vglut2)-positive glutamatergic neurons displayed CB1R-MOR co-expression. In contrast, only a small subset (5%–25%) of Vglut2-positive neurons in pain-related regions, such as the periaqueductal gray (PAG) and the dorsal horn of the spinal cord, or vesicular GABA transporter (Vgat)-positive GABA neurons in reward-related regions, such as the nucleus accumbens, ventral tegmental area, and substantia nigra, exhibited such colocalization. Unexpectedly, the selective deletion of MOR from Vglut2-positive glutamatergic neurons or Vgat-positive GABAergic neurons did not alter the effects of Δ^9^-tetrahydrocannabinol (Δ^9^-THC), including analgesia, hypothermia, catalepsy, rotarod locomotor impairment, or conditioned place aversion. Similarly, CB1R deletion from GABAergic neurons did not affect oxycodone-induced analgesia, hypothermia, or conditioned place preference. These findings do not support the hypothesis of a direct CB1R–MOR interaction. Instead, the enhanced analgesic effects of cannabinoids and opioids may result from the activation of CB1R and MOR in distinct neuronal populations or circuits.

## Introduction

Opioids are commonly used for relieving pain, but have significant abuse potential, causing over 100,000 overdose deaths annually and over 16 million active cases of opioid use disorder (OUD) [1]. Opioid action is mediated by activation of mu, delta, and kappa opioid receptors in the central nervous system. Current treatments for OUD, such as methadone and buprenorphine, also target mu and/or kappa opioid receptors and share similar unwanted side-effects, such as analgesic tolerance and dependence [2]. This has sparked significant research interest in developing non-opioid or safer opioid pharmacotherapies for OUD. Understanding how opioid receptors interact with other receptors to influence behavior and pain relief is crucial in advancing this effort.

With increasing cannabis legalization in the USA, cannabinoids have gained attention for their potential in treating chronic pain [3]. Opioids and cannabinoids produce effects such as analgesia, hypothermia, and reward or aversion [4–6], raising the question of whether their co-administration could enhance analgesia while reducing side-effects by lowering the required doses of each drug. Preclinical evidence supports this hypothesis. A recent meta-analysis of 19 preclinical studies found that 17 reported additive or synergistic analgesic effects from co-administering cannabinoids and opioids [7]. In addition, this combination has been shown to prevent opioid tolerance in analgesia [8–10]. Accordingly, a receptor interaction hypothesis was proposed to explain these effects. Pretreatment with opioid receptor antagonists has been reported to block Δ^9^-tetrahydroxycannabinoid (Δ^9^-THC)-induced analgesia and dopamine (DA) release in the nucleus accumbens (NAc) [11, 12]. Similarly, CB1 receptor antagonists have been reported to attenuate opioid analgesia [13, 14]. Findings from gene-knockout (KO) mice further support this hypothesis. CB1R deletion has been found to reduce opioid analgesia, conditioned place preference (CPP), and self-administration [15–18]. Likewise, mu opioid receptor (MOR) deletion has been reported to reduce Δ^9^-THC-induced conditioned place preference (CPP) or aversion (CPA), and withdrawal symptoms [17, 19]. However, conflicting findings have been reported in these and other studies [6, 15, 17, 19, 20]. Taken together, the data suggest a potential interaction between cannabinoid and opioid receptors, though further research is needed to clarify the mechanisms involved.

These interactions may be direct – through receptor heteromerization or signaling cross-talk downstream from receptor activation – or indirect via alteration in endogenous opioid peptide or endocannabinoid release, or by modulation of distinct neural circuits [5, 21–24]. Some in vitro studies support MOR-CB1R heteromerization in cultured cells [25, 26]. However, evidence demonstrating MOR-CB1R heterodimers in brain or spinal cord tissues remains sparse. Similarly, immunohistochemistry studies have suggested possible MOR and CB1R colocalization in rat dorsal horn interneurons [27], dorsal striatal neurons [28], and NAc neurons [28, 29]. However, clear proof of MOR-CB1R colocalization within the same neurons is still lacking, as MOR- and CB1R-immunostaining often overlap in different cells or interwoven nerve terminals, making precise distribution difficult to determine. Additionally, the specific neuronal phenotypes that co-express CB1R and MOR are not well defined. While both receptors are expressed in brain GABA and glutamate neurons [30–33], the proportion of these neurons co-expressing both receptors remains unknown. Moreover, the functional significance of such colocalization, if present, in the pharmacological effects of opioids and cannabinoids is largely unexplored.

In this study, we systematically evaluated the CB1R–MOR interaction hypothesis. Using RNAscope in situ hybridization, we first examined whether both receptor genes are co-expressed in GABAergic or glutamatergic neurons within pain- and reward-related brain regions in mice. We found that only a small subset of glutamate or GABA neurons co-expressed both CB1R and MOR in regions such as the periaqueductal gray (PAG), ventral tegmental area (VTA), NAc, and spinal dorsal horn. Next, we used Cre-LoxP techniques to selectively delete MOR or CB1R from Vgat-positive GABA or Vglut2-positive glutamate neurons and examined the behavioral responses of MOR-knockout (MOR-KO) mice to Δ^9^-THC and CB1R-KO mice to oxycodone. There are three main types of glutamatergic neurons in the brain, each defined by expression of a distinct vesicular glutamate transporter (Vglut) [34, 35]. Vglut1-positive neurons are primarily located in the cerebral cortex, hippocampus, and cerebellar cortex, where they support higher cognitive functions such as learning and memory. Vglut2-positive neurons are enriched in subcortical regions, including the thalamus, brainstem, and hypothalamus, and are involved in sensory processing, motivation, arousal, and pain regulation. In contrast, Vglut3 is expressed in a subset of atypical glutamatergic neurons, some serotonergic, GABAergic, and cholinergic cells, enabling glutamate co-release [36]. Given the critical roles of Vglut2-expressing neurons in the paraventricular thalamus (PVT), PAG, amygdala, and spinal cord in mood, reward, sensory integration, and substance use disorders [37–39], we focused our CB1R-MOR colocalization analysis on Vgat-positive GABAergic neurons and Vglut2-positive subcortical glutamatergic neurons. Our results showed that deletion of one receptor did not alter the response of the other to its ligand. These findings do not support the direct CB1R–MOR interaction hypothesis. Instead, we propose that the enhanced analgesic effects of cannabinoids and opioids may result from an indirect interaction between CB1R and MOR within distinct neuronal populations or circuits.

## Materials and methods

### Animals

Homozygous MOR-flox (B6;129-*Oprm1*^*tm1.1Cgrf*^/KffJ, Strain #:030074, the Jackson Laboratories) and CB1R-flox [32] mice were crossed with heterozygous Vglut2-Cre (Slc17a6^tm2(cre)Lowl^, stock #016963) or Vgat-Cre mice (*Slc32a1*^*tm2(cre)Lowl*^, stock #028862, The Jackson Laboratory, Bar Harbor, ME) at the National Institute on Drug Abuse (NIDA) Intramural Research Program (IRP) to generate conditional knockout mouse lines – Vglut2-MOR-KO (MOR-flox^+/+^ × Vglut2-Cre^+/^^−^) (Supplementary Fig. 1A), Vgat-MOR-KO (MOR-flox^+/+^ × Vgat-Cre^+/^^−^) (Supplementary Fig. 1B), Vgat-CB1-KO (CB1R-flox^+/+^ × Vgat-Cre^+/^^−^) (Supplementary Fig. 1C), and their “wild-type” littermates (MOR-flox^+/+^ × Vglut2-Cre^−^^/^^−^, MOR-flox^+/+^ × Vgat-Cre^−^^/^^−^, and CB1R-flox^+/+^ × Vgat-Cre^−^^/^^−^). Adult males and females (25–40 g, 6–9 months old) from these mouse lines were used in all experiments. Transgenic mice underwent genotyping by Transnetyx for verification. All subjects were kept on a 12-h reverse light cycle (lights off at 7:00 am; lights on at 7:00 pm) and provided with ad lib food and water. The house room temperature was set to 21–23 °C with 40%–50% humidity. Experimental procedures adhered to the Guide for the Care and Use of Laboratory Animals, 8th edition. The Animal Care and Use Committee at NIDA approved the study protocol.

### Chemicals

Oxycodone and Δ^9^-THC were provided by the NIDA Pharmacy (Baltimore, MD). Δ^9^-THC stock solution was dissolved in ethanol at a concentration of 20 mg/ml, and this stock solution was diluted as needed in a 5% Cremophor (Sigma-Aldrich, St. Louis, MO) saline solution. Oxycodone stock solution (50 mg/ml) was diluted in saline.

### Exp. 1: RNAScope in situ hybridization

RNAScope in situ hybridization was performed as we reported previously [40], aside from the specific probes and brain regions analyzed. Following sacrifice via rapid decapitation, mouse brains were wrapped in aluminum foil over dry ice and stored in a −80 °C freezer. For spinal cord extraction, the hydraulic extrusion protocol detailed by [41] was used, followed by storage in a −80 °C freezer until use. Sections from the cervical and lumbar regions of the spinal cord were collected for RNAScope. Coronal sections of brain tissue and axial sections of spinal cord tissue were taken on a cryostat at 16 μm thickness and mounted onto positively charged glass slides (Fisherbrand Superfrost Plus; Fisher Scientific, Pittsburgh, PA). For preparation, the slides were fixed in 10% cold formalin for 20 min, washed with PBS (×2), and dehydrated in graduated ethanol in three rounds from 50% to 100% EtOH. Next, slides were processed using the manufacturer’s protocol for the RNAscope Multiplex Fluorescent Reagent Kit v2 (Advanced Cell Diagnostics, Newark, CA). Hydrogen peroxide was applied to the slides for 10 min (room temperature), followed by dH2O washes (×2). Protease IV solution was then added to the slides for 30 min at room temperature, followed by PBS washes (x2). The appropriate mRNA probes were then added to the slides and hybridized in the HybEZ oven for 2 h at 40 °C, followed by 2 washes in the manufacturer-provided RNAscope wash buffer. Signal amplification steps followed, with AMP1 (30 min), AMP2 (30 min), and AMP3 (15 min) incubations at 40 °C, with RNAscope wash buffer (×2) applied after each step. To develop the fluorescent signal for each probe, horseradish peroxidase conjugates (*n*) [HRP-C(*n*)], where *n* is a given channel (1, 2, or 3), was added to the slides (15 min, 40 °C), followed by incubation with a diluted Opal dye of wavelength 520 nm, 570 nm, or 690 nm (30 min, 40 °C), with wash buffer applied after each step (×2). The following dilution ratios were utilized for each Opal dye: 1:1500 Opal 570, corresponding with *Cnr1* (CB1R) probe (Cat No. 420721), 1:1500 Opal 520, corresponding with *Slc17a6* (Vglut2) probe (Cat No. 319171-C3) or *Gad1* (GAD67) probe (Cat No. 400951-C3), and 1:1000 Opal 690, corresponding with Oprm1 probe (Cat No. 315841-C2). Slides were incubated with HRP blocker (15 min, 40 °C) and washed with buffer (×2) between each round of HRP-C(n) and Opal dye. DAPI was then applied to the slides (30 s, room temperature), and glass coverslips were added with fluorescent mounting medium (Fluoro-Gel; #17 985, Electron Microscopy Science). Images were taken at 4× and 40× magnification using a Keyence BZ-X800 Fluorescence Microscope.

#### Cell counting methods

RNAscope signals were analyzed at the cellular level using the Macro Cell Count function in the Keyence BZ-X software. DAPI staining was used to identify cell nuclei, and the software segmented individual cells based on nuclear position and cell morphology. Cells were classified as RNA-positive if the combined signal intensity within the cell exceeded a defined threshold. This threshold was chosen to reliably distinguish the true signal from the background while accounting for variability in signal intensity across cells. By evaluating expression at the whole-cell level rather than counting individual puncta, this approach provides a robust estimate of the percentage of cells expressing CB1 or MOR, particularly in brain regions with densely packed or overlapping neurons. This method reduces counting errors and captures biologically relevant expression patterns across neuronal populations.

Exposure settings for 40× images used for quantification were held constant across regions within each run of RNAscope and established based on visual inspection as the maximum exposure that did not over-expose the brightest areas. Across all runs, exposure varied from 1 to 2 s for the Cy5 channel (corresponding with *Oprm1* mRNA), 0.2–1.2 s for the GFP channel (corresponding with *Slc17a6*/*Gad1* mRNA), and 0.0167–0.167 s for the TRITC channel (corresponding with *Cnr1* mRNA). The Macro Cell Count application of the BZ-X800 Analyzer software was used for cell counting; briefly, thresholds for defining *Oprm1* (MOR), *Slc17a6* (Vglut2), *Gad1* (GAD67), *or Cnr1* (CB1R) area were established based on visual inspection and kept constant within runs of RNAscope. The level of nonspecific “noise” within the Cy5 channel varied within some RNAscope runs, so images were sorted into three levels of Cy5 noise, and the threshold was varied between the levels but kept the same within them. Standardizing exposure and cell counting threshold settings within RNAscope runs, across regions analyzed, allows for unbiased comparison of MOR or CB1R prevalence between regions. The threshold area of ≥1 µm^2^ was used to define CB1R^+^ only, MOR^+^ only, and (CB1R^+^ + MOR^+^) cells. The percentages of CB1R^+^ and/or MOR^+^ cells over Vglut2^+^ or GAD67^+^ cells were used for data analysis (*n* = 3 mice per group, with 1–2 male subjects or 1–2 female subjects for each brain region).

### Exp. 2: Δ^9^-THC-induced tetrad and oxycodone-induced analgesia and hypothermia

Tetrad measurements were performed as we reported previously [40]. MOR-flox mice, Vglut2-MOR-KO mice, and Vgat-MOR-KO mice (*n* = 7–8 per group, with 3–4 male and 3–4 female subjects per group) were treated with vehicle or Δ^9^-THC (10 or 30 mg/kg, i.p.) to measure cannabinoid-induced analgesia, catalepsy, rotarod motor impairment, and hypothermia. CB1R-flox mice and Vgat-CB1-KO mice (*n* = 7–8 per group, with 3–4 male and 3–4 female subjects per group) were treated with vehicle or oxycodone (3 or 10 mg/kg, i.p.) to measure opioid-induced analgesia and hypothermia. The order of testing was counterbalanced. Measurements were taken 0.5, 1, 2, and 3 h post-Δ^9^-THC or oxycodone injection on the testing day. Time intervals between test days were two to three days.

#### Analgesia

Measurement of hot-plate analgesia was performed as we previously reported [40]. Thermal nociception was measured prior to injections and 30 min after each injection using a hot-plate device (Model 39, IITC Life Science Inc., CA). Mice were placed on a hot-plate heated to 52 °C with a transparent barrier in place. The latency to exhibit the first thermal nociceptive sign, including paw licking, stomping, or shaking hind paws, and jumping, was recorded to the nearest hundredth of a second. Mice were removed from the hot-plate immediately after the first thermal nociceptive sign or, if no thermal nociceptive signs occurred, at 60 s to avoid tissue damage.

#### Hypothermia

To measure changes in body temperature, the RET-2 rectal probe (Harvard Apparatus, Holliston, MA) was lubricated with seed oil and gently inserted 2 cm into the rectum. Temperature was recorded once the measurement stabilized, to the nearest 0.1 °C.

#### Catalepsy

Cataleptic behavior was measured using an elevated bar test. Subjects’ front paws were placed on a metal bar at a height where their hind paws just reached the ground. The latency for the mice to remove both front paws from the bar and place them on the ground was recorded to the nearest tenth of a second, with a cutoff of 120 s.

#### Rotarod motor impairment

To measure mobility and coordination, a rotarod device (Harvard Apparatus), wherein subjects must walk on an elevated rotating rod, was used. Prior to testing, subjects underwent four days of training to establish a similar baseline, starting with a low speed of 10 revolutions per minute and increasing the speed to the point where each subject could stay on the rod at 20 rev/min for at least 1 min. The program used during tetrad testing gradually increased the rotarod speed from 4 rev/min to 40 rev/min over 5 min, and the latency for subjects to fall off the rod was measured to the nearest 0.1 second.

### Exp. 3: Conditioned place preference or aversion (CPP/CPA)

A classical CPP/CPA procedure was used to evaluate opioid-induced CPP [42–44] or Δ^9^-THC-induced CPA [45–47]. Subjects (*n* = 8–11, with equal or approximately equal numbers of male and female subjects) underwent a 7-day place conditioning regimen. During the preconditioning (days 1 and 2) and postconditioning (days 10 and 11) phases, mice were placed in the central corridor of a two-chambered apparatus and allowed to freely explore the apparatus for 15 min. The two chambers had distinct visual (black vs. white walls) and tactile (grate vs. bar floor) cues, and the amount of time spent in the corridor and each chamber was recorded to the nearest  0.1 second. Following preconditioning, one of the two chambers was designated as the drug-associated chamber, and the other was designated as the vehicle-associated chamber for each subject. Chambers were assigned to minimize the average preconditioning side bias, with balanced assignments to each chamber (i.e., the same number of subjects were drug conditioned in the black chamber as in the white chamber). On days 3–9, subjects were injected with the corresponding drug or vehicle, on alternating days, and immediately isolated in the designated chamber for 45 min. No subjects were excluded from analysis.

On drug days (days 3, 5, 7, 9), three groups of mice (MOR-flox, Vglut2-MOR-KO, and Vgat-MOR-KO) were injected intraperitoneally with Δ^9^-THC (5 mg/kg, i.p.), and two groups of mice (CB1R-flox and Vgat-CB1-KO) were injected with oxycodone (3 mg/kg, i.p.) and then immediately placed into one of the drug-assigned chambers. On vehicle treatment days (days 4, 6, 8), subjects received vehicle (5% Cremophor for THC, saline for oxycodone) and were immediately placed into the opposite chamber.

### Statistical analyses

All data are presented as means ± SEM. One-way or two-way repeated measures (RM) analyses of variance (ANOVA) were used to evaluate the effects of genotype and/or test compounds (oxycodone or Δ^9^-THC) on tetrad effects and CPP/CPA. Post-hoc group comparisons were conducted only if the ANOVA F value achieved *p* < 0.05. The value of *p* < 0.05 was used to indicate statistically significant differences among or between groups. Animal group sizes were chosen based on a power analysis (*n* ≥ 7 per group) and extensive previous experience with the animal models used. The group size is the number of independent values (individual animal), and statistical analysis was done using these independent values.

## Results

### CB1R and MOR expression and colocalization in the paraventricular nucleus of the thalamus (PVT)

**Fig. 1:**
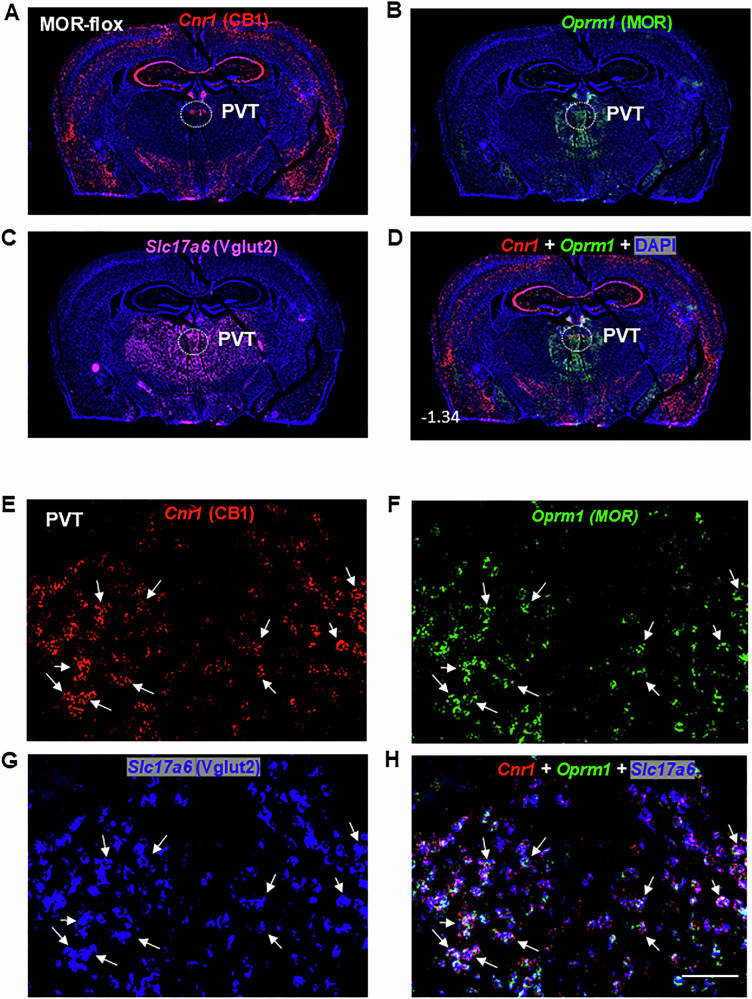
Regional and cellular distributions of CB1R and MOR mRNA, along with their colocalization in a brain section at Bregma level -1.34, assessed by RNAscope in situ hybridization in wild-type (MOR-flox and CB1-flox) mice. **A**–**D** Representative 4× images showing the mRNA expression of *Cnr1* (CB1R, red) (**A**), *Oprm1* (MOR, green) (**B**), and *Slc17a6* (Vglut2, magenta) (**C**), along with the overlaid *Cnr1* and *Oprm1* image (**D**). The images illustrate high-density CB1R expression in the cortex and hippocampus, and high-density MOR expression in the PVT, habenula, and around thalamic regions. Vglut2 is highly expressed in subcortical brain regions, particularly in the PVT and adjacent structures. **E**–**H** High magnification (40×) images, illustrating distinct cellular distributions of CB1R and MOR, as well as their colocalization in Vglut2-positive glutamate neurons of the PVT. *Cnr1* – Cannabinoid receptor 1; *Oprm1* – Mu opioid receptor 1; *Slc17a6* – Solute carrier family 17 member 6; PVT – paraventricular nucleus of the thalamus; DAPI – 4′,6-diamidino-2-phenylindole, a fluorescent stain that binds to adenine–thymine-rich regions of DNA. Scale bar: 50 μm.

Figure 1A, B illustrates the regional distributions of CB1R and MOR mRNA in the brain (at Bregma level −1.34) at 4× magnification, highlighting that *Cnr1* (CB1R) is more widely and abundantly distributed than *Oprm1* (MOR). High densities of CB1R mRNA were observed in the cerebral cortex and hippocampus, whereas MOR was prominently expressed in the subcortical paraventricular nucleus of the thalamus (PVT), surrounding thalamic areas, and the habenula. Figure 1C shows the distribution of *Slc17a6* (Vglut2^+^) glutamatergic neurons within the same coronal section, illustrating that these neurons are primarily expressed in subcortical regions, including the PVT and surrounding thalamic areas. Figure 1D presents an overlaid image of CB1R and MOR mRNA, further demonstrating their distinct regional distributions.

**Fig. 2:**
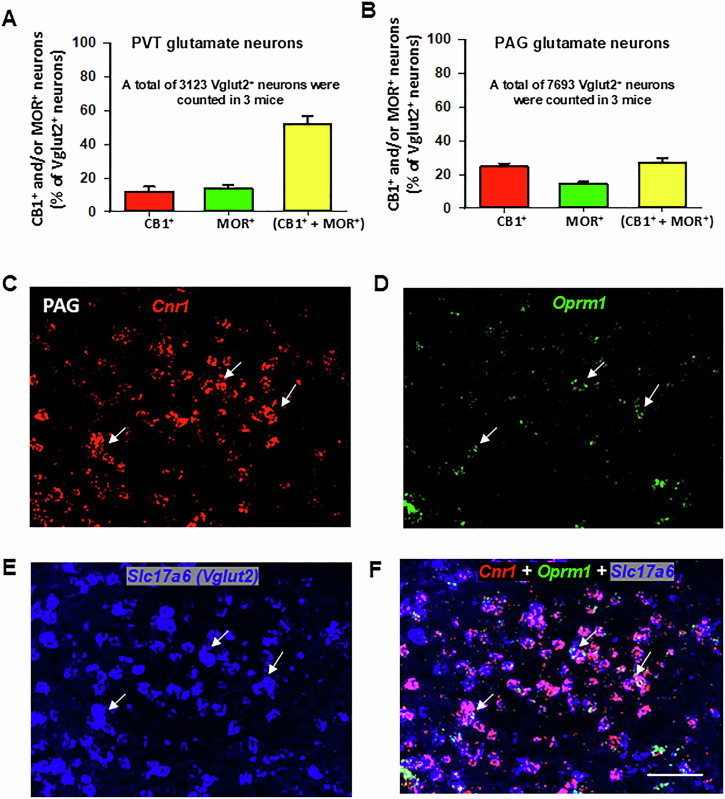
Glutamatergic CB1R and MOR expression and their colocalization in the PVT and PAG of wild-type (MOR-flox and CB1-flox) mice. **A**, **B** Quantitative cell counting results showing that ~50% (51.57 ± 5.33%) of Vglut2^+^ glutamate neurons in the PVT (**A**) and ~25% (27.16 ± 2.29%) in the PAG (**B**) co-express CB1R and MOR. In the PVT, ~12% of Vglut2^+^ neurons express CB1R and ~14% express MOR alone, whereas in the PAG, about 25% of Vglut2^+^ neurons express CB1R and ~15% express MOR alone. **C**–**E** Representative high-magnification (40×) images illustrating *Cnr1* (**C**, red), *Oprm1* (**D**, green), and *Slc17a6* (**E**, blue) in the PAG. **F** Overlay image showing that a small subset of Vglut2^+^ glutamate neurons co-express CB1R and MOR mRNA (highlighted by white arrows). Scale bar: 50 μm.

Figure 1E–H display representative high-magnification (40×) images of *Cnr1*, *Oprm1*, *Slc17a6*, and their overlay in the PVT of wild-type (MOR-flox and CB1-flox) mice. Among Vglut2-positive glutamate neurons in the PVT, approximately 12% (±3%) expressed *Cnr1* only, 14% (±2%) expressed *Oprm1* only, and 52% (±5%) co-expressed both *Cnr1* and *Oprm1* (Fig. 2A).

### CB1R and MOR expression and colocalization in the periaqueductal gray (PAG) and spinal cord

Next, we examined whether CB1R-MOR colocalization occurs in Vglut2^+^ glutamate neurons in the PAG and dorsal horn of the spinal cord – critical regions involved in pain processing and analgesia [48, 49]. Figure 2B shows that only a small subset (27% ± 2%) of Vglut2^+^ glutamate neurons co-expressed CB1R and MOR. Figure 2C–F shows representative high-magnification images, illustrating expression of glutamatergic *Cnr1* and *Oprm1* in the PAG at the Bregma level of −3.80. This finding aligns with a previous report indicating that ~32% of PAG Nissl-stained cells co-expressed CB1R and MOR immunostaining [50], though the neuronal phenotype was unidentified in that study.

**Fig. 3:**
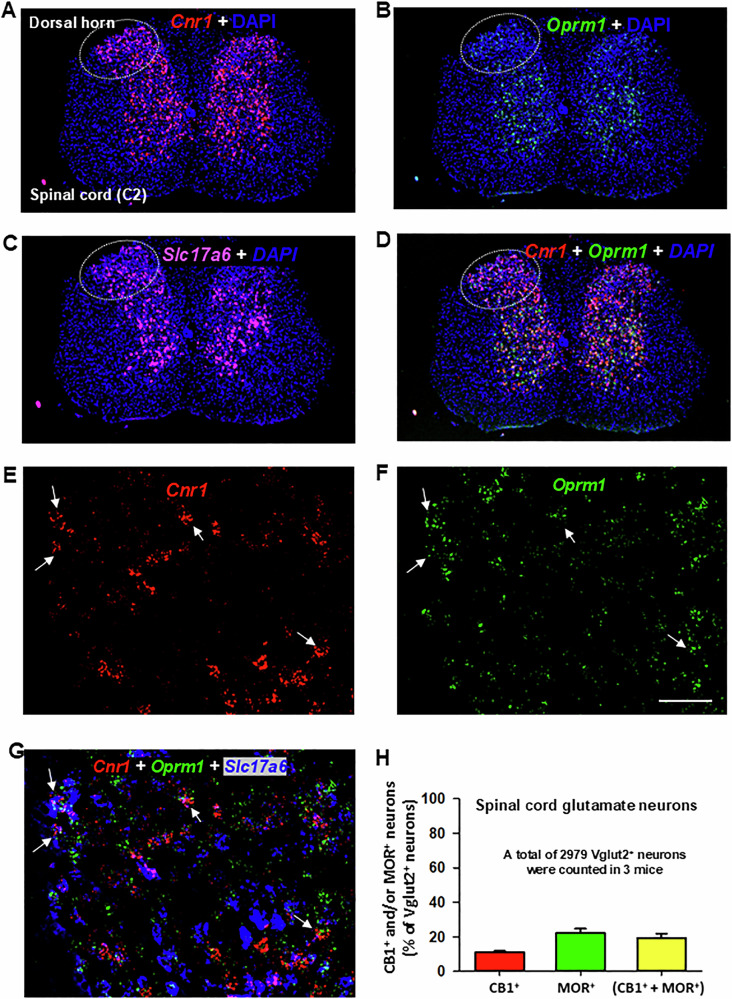
Glutamatergic CB1R and MOR expression and their colocalization in the spinal cord. **A**–**D** Representative 4× images showing the mRNA expression of *Cnr1* (red) (**A**), *Oprm1* (green) (**B**), and *Slc17a6* (magenta) (**C**), along with the overlay of *Cnr1* and *Oprm1* (**D**). The images illustrate a high density of CB1R and a low density of MOR expression in the spinal dorsal horn. Notably, Vglut2 is also highly expressed in the spinal cord. **E**–**G** High-magnification (40×) images, illustrating the distinct cellular distributions of CB1R and MOR, as well as their colocalization in a small population of Vglut2-positive glutamate neurons. **H** Quantitative cell counting data show that ~20% of Vglut2^+^ glutamate neurons in the spinal dorsal horn co-express CB1R and MOR. Scale bar: 50 μm.

Figure 3 illustrates CB1R and MOR mRNA expression and their colocalization in the dorsal horn of the spinal cord under low-magnification (Fig. 3A–D) and high-magnification (Fig. 3E–G). High densities of *Cnr1* and *Oprm1*, particularly *Cnr1*, were observed in this region. Notably, both receptors exhibited distinct cellular distributions (Fig. 3D), with the majority of CB1R and MOR expression occurring in non-glutamatergic (Vglut2^−^) cells (Fig. 3G). Only a small proportion of Vglut2^+^ glutamate neurons in the dorsal horn co-expressed *Cnr1* and *Oprm1* (19% ± 2%) (Fig. 3H).

### CB1R and MOR expression and colocalization in the NAc, VTA, and SNr

We then examined CB1R, MOR, and their colocalization in brain reward-related regions, including the nucleus accumbens (NAc), ventral tegmental area (VTA), and substantia nigra pars reticulata (SNr). Given the critical role of GABAergic neurons in drug reward and aversion [33, 46, 51], we analyzed CB1R-MOR co-expression in glutamate decarboxylase 1 (*Gad1*)^+^ GABA neurons in these regions.

Supplementary Fig. 2 shows *Cnr1* and *Oprm1* expression in *Gad1*^*+*^ GABA neurons in the NAc (Bregma level of 1.34) under low (Supplementary Fig. 2A) and high magnification (40×) (Supplementary Fig. 2C–F), highlighting their distinct regional distributions. *Cnr1* (red) was highly expressed in the dorsolateral striatum, whereas *Oprm1* (green) was predominantly expressed in the ventral striatum (NAc). Cell counting indicated that 4% (±2%) of *Gad1*^*+*^ GABA neurons expressed *Cnr1* only, 34% (±7%) expressed *Oprm1* only, and 12% (±3%) co-expressed both *Cnr1* and *Oprm1* in the NAc (Supplementary Fig. 2B).

We also examined *Cnr1* and *Oprm1* expression in *Gad1*^*+*^ GABA neurons in the VTA (Supplementary Fig. 3) and SNr (Supplementary Fig. 4). Overall, both receptors were expressed at low levels in these midbrain regions. Quantitative cell counting revealed that 17% (±2%) of GABA neurons in the VTA (Supplementary Fig. 3B) and 21% (±4%) of GABA neurons in the SNr (Supplementary Fig. 4B) co-expressed both receptors.

### Validation of mRNA signal specificity

To assess the specificity of our RNAscope assay, we used conditional MOR-KO mice. In the PVT, *Oprm1* (green) was largely absent from *Slc17a6*^*+*^ glutamate neurons in Vglut2-MOR-KO mice (Supplementary Fig. 5A) compared to MOR-flox littermates (Fig. 1F–H). However, *Cnr*1 expression in glutamatergic neurons remained unchanged in Vglut2-MOR-KO mice (Supplementary Fig. 5B) relative to wild-type controls (Fig. 1E, G, H). Quantitative cell counting revealed a significant reduction in the percentage of *Slc17a6*^*+*^ glutamate neurons expressing MOR in the PVT, from 65% ± 6% in MOR-flox mice to 32% ± 14% in Vglut2-MOR-KO mice (Supplementary Fig. 5E, *p < 0.05).

Similarly, we examined *Oprm1* expression in *Gad1*^*+*^ GABA neurons in MOR-flox and Vgat-MOR-KO mice (Supplementary Fig. 5C, D). Significant *Oprm1* expression was detected in GAD1^+^ GABA neurons in the NAc of MOR-flox (control) mice (Supplementary Fig. 2F), but not in Vgat-MOR-KO mice (Supplementary Fig. 5C). Cell counting indicated a significant reduction in *Oprm1* expression in GABA neurons of Vgat-MOR-KO mice (22% ± 12%) compared to 46% ± 4% in MOR-flox littermates (Supplementary Fig. 5F, *p < 0.05).

### Effects of MOR conditional knockout on the Δ^9^-THC-induced tetrad effects

**Fig. 4:**
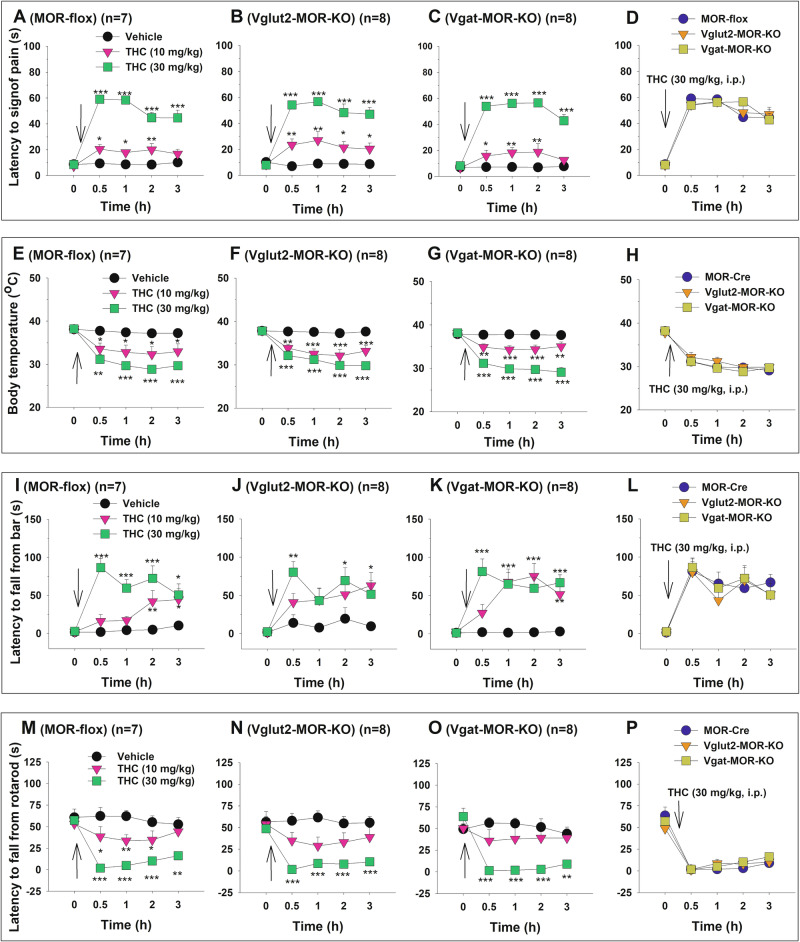
Δ^9^-THC-induced tetrad effects in conditional MOR-KO mice and their wild-type (MOR-flox) littermates. **A**–**C** Dose-dependent analgesic effects of Δ^9^-THC (0, 10, 30 mg/kg, i.p.) observed in MOR-flox (**A**), Vglut2-MOR-KO (**B**), and Vgat-MOR-KO (**C**) mice. **D** Analgesic effects of 30 mg/kg THC across the three genotypes, showing no significant genotype differences in Δ^9^-THC-induced analgesia. **E**–**G** Dose-dependent hypothermic effects of Δ^9^-THC (0, 10, 30 mg/kg, i.p.) observed in MOR-flox (**E**), Vglut2-MOR-KO (**F**), and Vgat-MOR-KO (**G**) mice. **H** Hypothermic effects of 30 mg/kg THC across the three genotypes, showing no significant genotype differences in THC-induced hypothermia. **I**–**K** Dose-dependent cataleptic effects of Δ^9^-THC (0, 10, 30 mg/kg, i.p.) observed in MOR-flox (**I**), Vglut2-MOR-KO (**J**), and Vgat-MOR-KO (**K**) mice. **L** Cataleptic effects of 30 mg/kg THC across the three genotypes, showing no significant genotype differences in THC-induced catalepsy. **M**–**O** Dose-dependent effects of Δ^9^-THC (0, 10, 30 mg/kg, i.p.) on rotarod locomotor performance observed in MOR-flox (**M**), Vglut2-MOR-KO (**N**), and Vgat-MOR-KO (**O**) mice. **P** Locomotor impairment induced by 30 mg/kg THC across the three genotypes, showing no significant genotype differences in THC-induced locomotor impairment. *p < 0.05, **p < 0.01, ***p < 0.001, compared to vehicle at each time point. *n* = 7 MOR-flox mice, 8 Vglut2-MOR-KO mice, and 8 Vgat-MOR-KO mice.

To assess the functional significance of CB1R and MOR colocalization, we examined the effects of conditional CB1R or MOR knockout on classical behavioral and physiological responses to cannabinoids and opioids, respectively. Systemic administration of Δ^9^-THC produced dose-dependent tetrad effects across all three genotypes – MOR-flox (Fig. 4A, E, I, M), Vglut2-MOR-KO (Fig. 4B, F, J. N), and Vgat-MOR-KO (Fig. 4C, G, K, O). A two-way repeated measures (RM) ANOVA revealed significant main effects of Δ^9^-THC dose, time, and dose × time interaction across all genotypes in analgesia (Fig. 4A–C), body temperature (Fig. 4E–G), catalepsy (Fig. 4I–K), and rotarod locomotor performance (Fig. 4M–O) (see Supplementary Table 1 for detailed *F* and *p* values).

However, when Δ^9^-THC effects were compared across genotypes in the same figure, no significant genotype differences were observed in analgesia (Fig. 4D), hypothermia (Fig. 4H), catalepsy (Fig. 4L), or rotarod performance (Fig. 4P). Detailed statistical results for each panel are provided in Supplementary Table 1. These findings suggest that selective MOR deletion from either GABA or glutamate neurons does not affect the classical pharmacological effects of cannabinoids.

### Effects of conditional CB1R knockout on oxycodone-induced analgesia and hypothermia

**Fig. 5:**
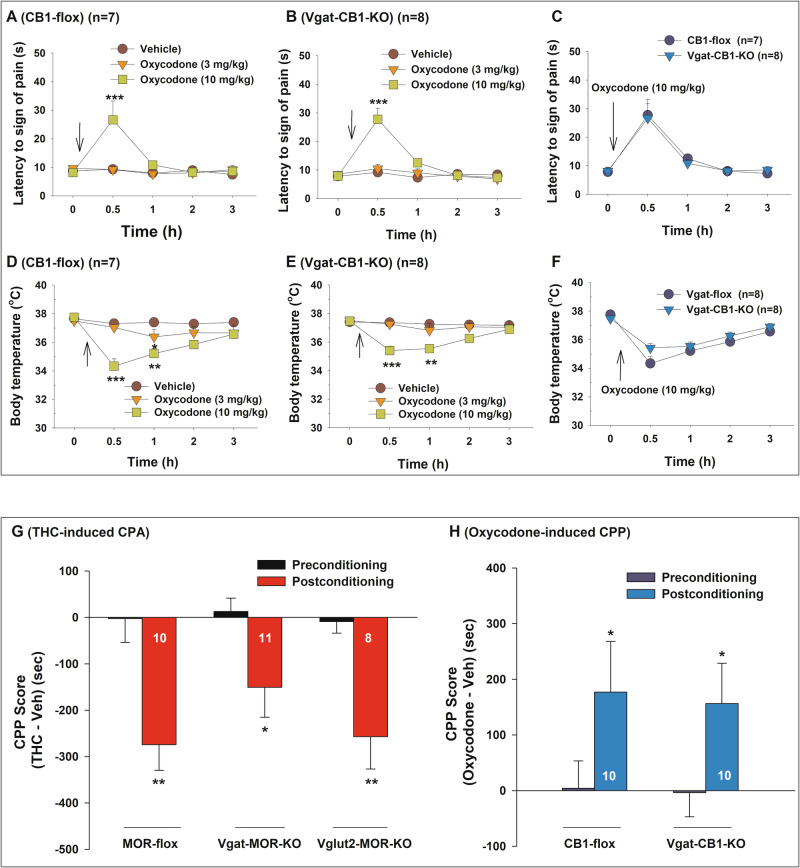
Effects of conditional CB1R or MOR knockout on oxycodone-induced analgesia and hypothermia, as well as the CPP/CPA response to Δ^9^-THC or oxycodone. **A**–**B** Dose-dependent effects of oxycodone (0, 3, 10 mg/kg, i.p.) on hot-plate analgesia in CB1-flox (**A**) and Vgat-CB1-KO (**B**) mice. **C** Analgesic effects of 10 mg/kg oxycodone across the two genotypes, showing no significant genotype differences in opioid-induced analgesia. **D**–**E** Dose-dependent effects of oxycodone (0, 3, 10 mg/kg, i.p.) on body temperature in CB1-flox (**D**) and Vgat-CB1-KO (**E**) mice. **F** Hypothermic effects of 10 mg/kg oxycodone across the two genotypes, showing no significant genotype differences in opioid-induced hypothermia. **G** Δ^9^-THC (5 mg/kg, i.p., for 4 days of conditioning) produced significant CPA in MOR-flox, Vgat-MOR-KO, and Vglut2-MOR-KO mice. **H** Oxycodone (3 mg/kg, i.p., for 5 days of conditioning) produced a similar CPP response in both CB1-flox control and Vgat-CB1-KO mice. *p < 0.05, **p < 0.01, com*p*ared to preconditioning. *n* = 7 CB1-flox mice a*n*d 8 Vgat-CB1-KO mice for catalepsy/analgesia experiments (**A**–**F**). *n* = 8–11 mice for co*n*ditioned place preference experiments (**G**, **H**, see numbers on bars).

We next examined the effects of conditional CB1R knockout from GABA neurons on opioid-induced analgesia and hypothermia. Systemic oxycodone administration (0, 3, 10 mg/kg, i.p.) produced a dose-dependent increase in hot-plate analgesia (Fig. 5A, B) and a dose-dependent decrease in body temperature (Fig. 5D, E) in both CB1-flox (control) and Vgat-CB1-KO mice. A two-way RM ANOVA revealed significant main effects of oxycodone, time, and treatment × time interaction (see Supplementary Table 2 for *F* and *p* values).

However, when oxycodone effects were compared between CB1-flox and Vgat-CB1-KO mice (Fig. 5C, F), no significant genotype main effect or genotype × time interaction was detected (Supplementary Table 2). These results suggest that selective CB1R deletion from GABA neurons does not alter opioid-induced behavioral and physiological responses.

### Effects of conditional deletion of MOR or CB1R on CPP response to Δ^9^-THC or oxycodone

Finally, we investigated whether conditional MOR or CB1R knockout affects conditioned place preference (CPP) or conditioned place aversion (CPA) in response to oxycodone or Δ^9^-THC. Systemic Δ^9^-THC administration (5 mg/kg) induced a strong CPA response in all three strains of mice, with no significant differences in CPA responses between MOR-flox, Vglut2-MOR-KO, and Vgat-MOR-KO mice (Fig. 5G). A two-way RM ANOVA revealed a significant main effect of Δ^9^-THC conditioning (*F*_1, 26_ = 32.57, *p* < 0.001), but no significant genotype main effect (*F*_2, 26__ = 0.8130, *p* = 0.4545) or genotype × conditioning interaction (*F*_2, 26_ = 0.9845, *p* = 0.387). Post-hoc comparisons confirmed that Δ^9^-THC-induced CPA was significant in all genotypes compared to preconditioning (Fig. 5G). These findings suggest that MOR deletion from either GABA or glutamate neurons does not alter Δ^9^-THC-induced CPA.

We also examined the effects of CB1R deletion from GABA neurons on oxycodone-induced CPP (Fig. 5H). Oxycodone (3 mg/kg) produced significant CPP in both CB1R-flox and Vgat-CB1-KO mice. A two-way RM ANOVA revealed a significant conditioning main effect (*F*_1, 18_ = 9.387, *p* = 0.007), but no significant main effect of genotype (*F*_1, 18_ = 0.0190, *p* = 0.892) or genotype × conditioning interaction (*F*_1, 18_ = 0.0327, *p* = 0.859). Post-hoc group comparisons indicate that oxycodone-induced CPP is significant in CB1R-flox (*p < 0.05) and Vgat-CB1-KO (*p* = 0.049) mice, compared to preconditioning.

## Discussion

This study examined the CB1R–MOR interaction hypothesis using RNAscope ISH and behavioral tests in both wild-type and conditional CB1R- or MOR-KO mice. We found distinct regional distributions of CB1R and MOR, with limited colocalization (5%–25%) in *Vglut2*^*+*^ glutamate or *Vgat*^*+*^ GABA neurons in pain- and reward-associated brain regions, including the PAG, spinal dorsal horn, NAc, VTA, and SNr). An exception was the PVT, where ~50% of *Vglut2*^*+*^ neurons co-expressed CB1R and MOR. Selective deletion of MOR from Vgat^+^ GABA or Vglut2^+^ glutamate neurons did not alter Δ^9^-THC-induced tetrad effects and CPA, nor did CB1R deletion from GABA neurons affect oxycodone-induced analgesia, hypothermia, and CPP. These findings do not support a direct CB1R–MOR interaction hypothesis but rather point to a possible indirect interaction that may be responsible for cannabinoid-opioid enhancement of analgesia.

### Opioid and cannabinoid interactions in pain and analgesia

Preclinical studies suggest functional interactions between opioid and cannabinoid systems, as cannabinoid agonists enhance opioid analgesia, reducing required doses and extending analgesic duration [4, 5, 7, 24]. These effects are often labeled “synergistic” [52–57], but most studies lack quantitative “additive” vs. “synergistic” analyses. Linear isobolographic analysis, a well-established method for this purpose [58–60], has shown mixed results: synergistic [61, 62] or antagonistic [63] effects of opioid agonist and cannabinoid agonist co-administration. Human laboratory studies and clinical trials have failed to confirm significant opioid-sparing or analgesia-enhancing effects from cannabinoid-opioid co-administration [57].

Using conditional MOR-KO and CB1-KO mice, we found that MOR deletion from GABA or glutamate neurons did not affect Δ^9^-THC’s analgesic, hypothermic, cataleptic, or rotarod locomotor effects. Likewise, CB1R deletion from GABA neurons did not alter opioid analgesia, hypothermia, and CPP. These results highlight the complexity of cannabinoid–opioid interactions, which may vary depending on experimental conditions.

### Opioid and cannabinoid interactions in drug reward and aversion

We also found that deleting MOR from GABA or glutamate neurons did not affect Δ^9^-THC-induced aversion, nor did CB1R deletion from GABA neurons alter oxycodone-induced CPP. This suggests that CB1R and MOR function independently in cannabinoid- and opioid-induced reward. However, previous studies report reciprocal enhancement of cannabinoid and opioid rewarding effects. For instance, cannabinoids (Δ^9^-THC, CP55940, WIN55212-2) increase morphine or heroin intake [64–68], while CB1R deletion or pharmacological blockade reduces opioid self-administration [16, 18, 69–72]. CB1R activation in the VTA or NAc enhances morphine-induced CPP [73], whereas CB1R blockade attenuates it [15, 74–77]. In brain microdialysis experiments, Δ^9^-THC increases NAc extracellular DA, an effect blocked by naloxone [11]. Similarly, CB1R deletion prevented the morphine-induced DA release [78].

However, not all evidence supports the above findings. Some studies show attenuation of opioid reward by cannabinoids or vice versa. Δ^9^-THC was reported to reduce oxycodone self-administration [66, 79, 80], and CB1R-KO mice showed normal morphine-induced CPP [20]. CB1R blockade did not affect heroin-induced NAc DA release [69] or morphine-induced locomotor sensitization [81]. Furthermore, it was reported that CB1R activation in the basolateral amygdala switched morphine’s rewarding effects to aversion, while CB1R blockade enhanced morphine-induced CPP [82]. These findings indicate that cannabinoid–opioid interactions in drug reward are more complex than previously assumed. Our results in conditional KO mice do not support a direct CB1R–MOR interaction that occurs in GABA or subcortical glutamate neurons.

### Opioid and cannabinoid interactions at the receptor level

The mechanisms underlying opioid-cannabinoid interactions remain unclear. Hypotheses include CB1R-MOR heterodimerization, allosteric modulation, competition for downstream signaling molecules, or modulation of endogenous opioid or endocannabinoid release [5, 21–24].

There is in vitro evidence for a direct CB1R–MOR interaction; BRET/FRET assays in co-transfected HEK and BHK cells have supported receptor heteromerization [25, 26]. Previous immunohistology studies have also suggested possible CB1R-MOR colocalization in the dorsal horn, PAG, dorsal striatum, and NAc [27, 29]. CB1R and MOR may also compete for Gi/o-proteins in co-transfected cells [83] and interact via an allosteric modulation in ligand binding [84]. However, there is a lack of evidence supporting the presence of CB1R-MOR heterodimers in brain tissues in vivo. Furthermore, it remains unknown which neuronal phenotypes co-express CB1R and MOR or whether such heterodimers or co-expression play a functional role in the actions mediated by cannabinoids or opioids. To test this hypothesis, we proposed that if CB1R and MOR interact directly and functionally, both receptor genes should be expressed in the same GABAergic or glutamatergic neurons. Additionally, the deletion of one receptor should alter the response of the other receptor to its ligand and vice versa.

Using RNAscope ISH, we quantified CB1R and MOR mRNA expression in pain- and reward-related brain regions. *Cnr1* and *Oprm1* mRNA had distinct distributions, with limited (5%–25%) colocalization in GABA and glutamate neurons in the PAG, spinal dorsal horn, NAc, VTA, and SNr. Consistent with this finding, distinct CB1R and MOR distributions have also been observed in the brainstem, where MOR expression is high but CB1R expression is minimal [85–87]. These findings suggest that CB1R and MOR may function independently from each other.

Notably, the ~50% colocalization of CB1R and MOR in PVT glutamatergic neurons suggests a potential interaction on a molecular level [25]. Chemogenetic and optogenetic studies have shown that manipulation of PVT glutamatergic neurons alters both pain-like and reward-seeking behaviors, implicating this population in chronic pain, opioid analgesia, and addiction-related processes [37, 88]. However, our finding that selective deletion of MOR from Vglut2^+^ neurons – including those in the PVT – does not affect Δ9-THC-induced behavioral responses in tetrad or CPP/CPA assays suggests that CB1R–MOR interaction in PVT glutamate neurons may not play a major role in mediating cannabinoid effects.

### Opioid and cannabinoid interactions by indirect mechanisms

CB1R and MOR may also interact indirectly by modulating endogenous opioid or endocannabinoid levels. Δ^9^-THC increases opioid peptide gene expression [89, 90]. Opioid administration alters AEA and 2-AG levels in the striatum, limbic forebrain, and hippocampus [91, 92], and heroin self-administration increases extracellular AEA while decreasing 2-AG in the NAc [93]. Chronic opioid use may modify CB1R expression [19, 91], though findings are inconsistent [94, 95]. These data suggest a subtle modulatory role of cannabinoids on opioids and vice versa, rather than a direct CB1R–MOR interaction.

### Limitations of the study

This study has several limitations. First, we used pan-GABA Cre (Vgat-Cre) mice and Vglut2-Cre mice to investigate the function of CB1R-MOR colocalization in broad GABAergic neurons and subcortical glutamatergic populations. However, this approach did not target MOR expression in Vglut1- or Vglut3-expressing glutamatergic neurons. Therefore, we cannot exclude the possibility of CB1R-MOR colocalization within these glutamatergic subpopulations, nor their potential involvement in cannabinoid–opioid interactions. Since there is no pan-glutamate Cre line available, we plan to generate Vglut1/2 double-Cre mice to enable broader glutamatergic targeting. Second, our experiments used Vgat-CB1-KO mice but not Vglut2-CB1-KO mice. Although CB1Rs have been shown to primarily exert their effects via GABA neurons [96, 97], a potential role for glutamate neuronal CB1Rs in cannabinoid–opioid interactions cannot be excluded [32]. Third, although our findings do not support a direct interaction between CB1R and MOR at the receptor level, we cannot rule out the possibility that other opioid receptors (e.g., delta or kappa) and other cannabinoid receptors (e.g., CB2) may form functional heterodimers and contribute to cannabinoid–opioid interactions. Fourth, we did not include additional positive control experiments to demonstrate whether conditional deletion of CB1 or MOR from GABA or glutamate neurons alters cannabinoid or opioid actions, as such findings have been reported previously [32, 46], and further data will be published separately. Importantly, the absence of these additional controls does not affect the main conclusions of this study. Fifth, the percentage of mouse SNr GABA neurons expressing MOR in the present work (32%) is lower than previously reported in rats (46%) [33], which may reflect species differences, use of distinct RNAscope probes, or methodological differences in quantitative cell counting. Last, CB1Rs and MORs were constitutively deleted during development, which may have led to compensatory changes in unaffected neurons, potentially masking interactions between these receptors.

In summary, this study systematically evaluated CB1R–MOR interactions at receptor, signaling, and behavioral levels. CB1R and MOR showed distinct regional and cellular distributions, with limited colocalization in GABA or glutamate neurons in pain- and reward-related areas. Conditional deletion of MOR from GABA or glutamate neurons did not alter Δ^9^-THC-induced tetrad effects or CPA, nor did CB1R deletion from GABA neurons affect oxycodone-induced analgesia, hypothermia, or CPP. These findings do not support a CB1R-MOR heterodimer or direct interaction, but rather point to a more subtle indirect, modulatory relationship between CB1R and MOR.

## Supplementary information

Supplementary Materials

## Data Availability

All the data are presented in the main manuscript and the additional supporting files. They will be deposited in a publicly available repository (GitHub) after the paper is accepted for publication by the NIH Data Management and Sharing (DMS) policy.
