# Supplementary material for: Cannabinoid CB1 receptor and mu-opioid receptor interaction: new insights from conditional knockout mice
Source: Neuropsychopharmacology. 2025 Sep 25;51(2):506–18. doi: 10.1038/s41386-025-02245-6 (PMC12708859; doi:10.1038/s41386-025-02245-6)
Supplement: Supplementary file 1 — Supplementary Materials [file 41386_2025_2245_MOESM1_ESM.docx]

Neuropsychopharmacology

**Cannabinoid CB1 receptor and mu-opioid receptor interaction: New insights from conditional knockout mice**

Hannah Alton, Emily Linz, Guo-Hua Bi, Omar Soler-Cedeno, Maia Maras, Zheng-Xiong Xi^#^

**Supplementary Information**

**Supplementary Figure 1:**

**
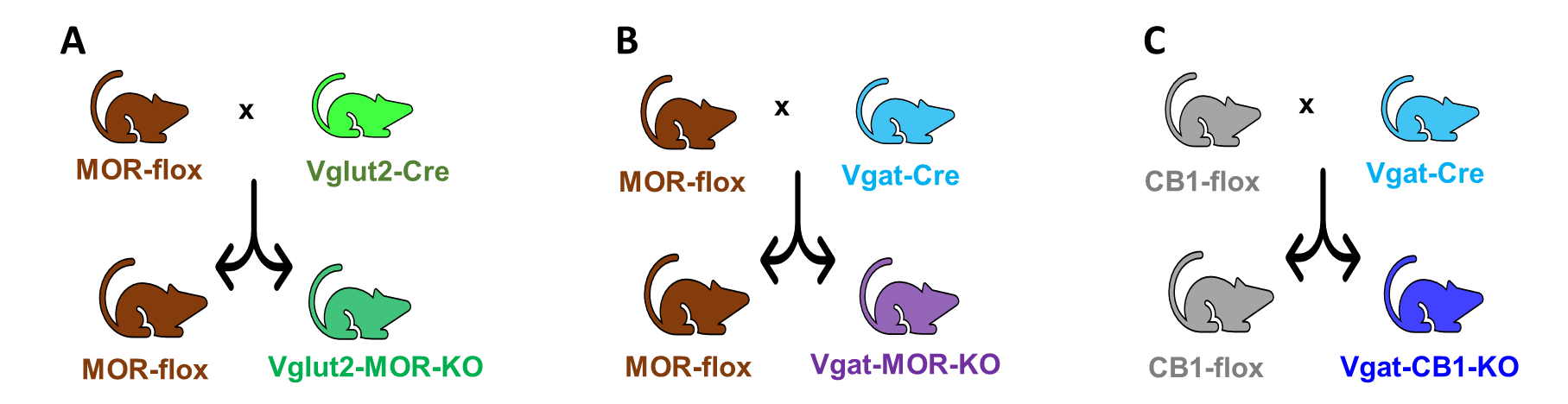
**

**Supplementary Figure 1**: The Cre-loxP strategies used to generate conditional MOR-KO mice from Vglut2^+^ glutamate neurons (**A**), Vgat^+^ GABA neurons (**B**), or conditional CB1-KO from Vgat^+^ GABA neurons (**C**).

**Supplemtary Figure 2:**

**
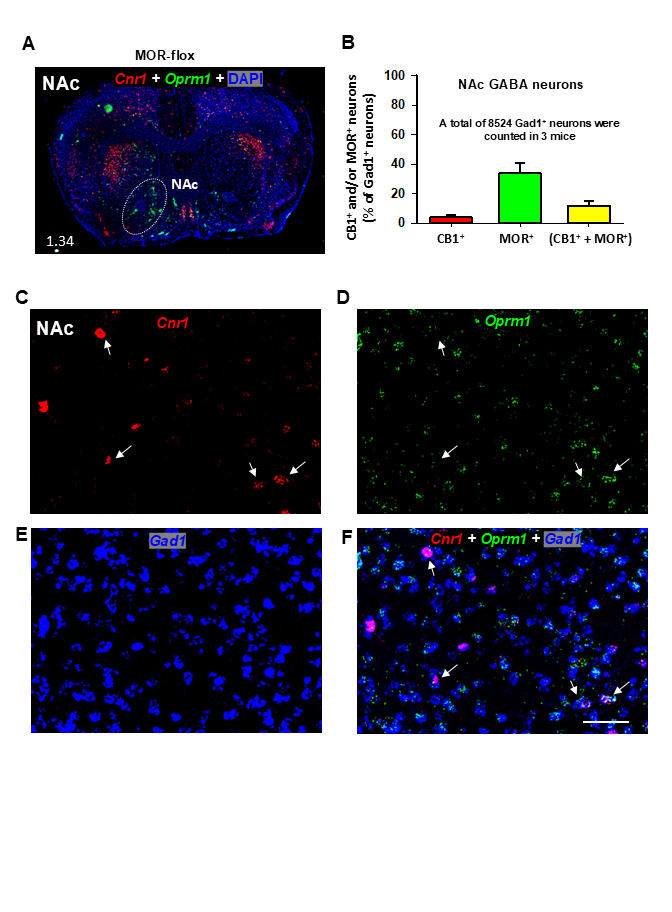
**

**Supplementary Figure 2:** GABAergic CB1R and MOR expression and their colocalization in the NAc of MOR-flox and CB1-flox mice. **A**: Overlaid images (4×) of *Cnr1* and *Oprm1* mRNA in a brain slice at Bregma level of 1.34 mm, illustrating high-density MOR and low density CB1R expression in the NAc. **B**: Quantitative cell counting data reveal that <5% of *Gad1*^+^ GABA neurons express CB1R alone, 34% express MOR alone, and 12% co-express CB1R and MOR. The number shown in the bar figure represents the total number of *Gad1*^+^ GABAergic neurons analyzed from the NAc, from which percentages of CB1R^+^ and/or MOR^+^ neurons were calculated. **C**-**F**: Representative high-magnification (40×) images, illustrating a small subset of *Gad1^+^* GABA neurons co-express CB1R and MOR in the NAc. NAc – nucleus accumbens; *Gad1* – glutamate decarboxylase 1 (a GABA neuronal marker). Scale bar: 50 μm.

**Supplementary Figure 3:**

**
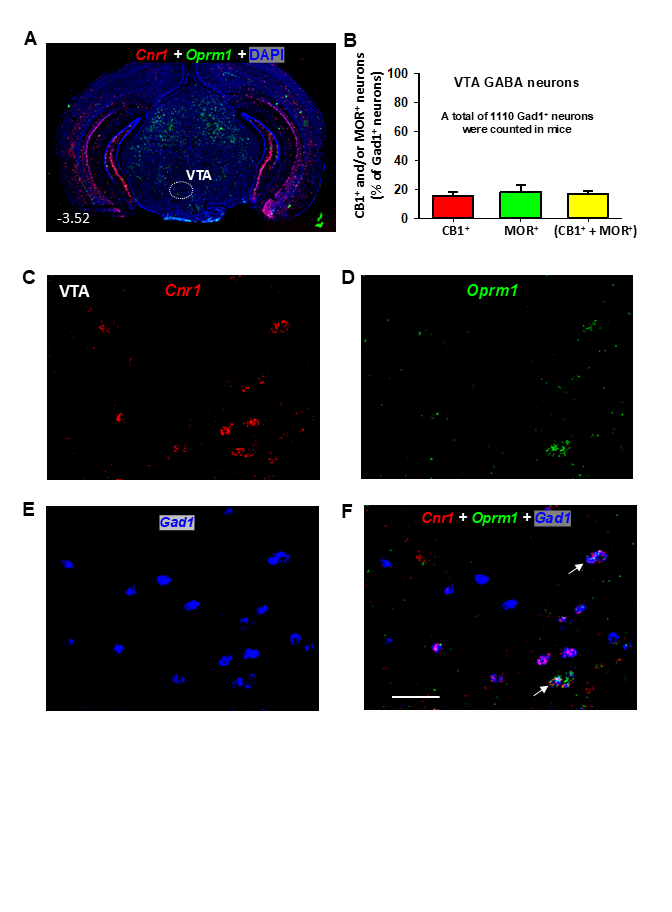
**

**Supplementary Figure 3**: GABAergic CB1R and MOR expression and colocalization in the VTA of MOR-flox and CB1-flox mice. **A**: Overlaid 4× images of Cnr1 and Oprm1 mRNA in brain slices at the Bregma level of -3.52 mm, illustrating low-density CB1R and MOR expression in the VTA. **B**: Quantitative cell counting data reveal that ~20% of *Gad1*^+^ GABA neurons express CB1R or MOR alone, and ~20% co-express CB1R and MOR. The number shown in the bar figure represents the total number of *Gad1*^+^ GABAergic neurons analyzed from the VTA, from which percentages of CB1R^+^ and/or MOR^+^ neurons were calculated. **C**-**F**: Representative high-magnification (40×) images, illustrating that a small subset of *Gad1^+^* GABA neurons co-express CB1R and MOR in the VTA. VTA – ventral tegmental area; *Gad1* – glutamate decarboxylase 1 (a GABA neuronal marker). Scale bar: 50 μm.

**Supplementary Table 1:** Statistical results of THC-induced tetrad effects in wildtype littermates (MOR-flox), Vgat-MOR-KO, and Vglut2-MOR-KO mice as shown in Figure 4.

| Measurement | Figure 4 | Main effects | Statistical result |
| --- | --- | --- | --- |
| Analgesia | A | Dose main effect | F_2,12_=67.127, p<0.001 |
|  |  | Time main effect | F_4,24_=36.342, p<0.001 |
|  |  | Interaction | F_8,48_=22.904, p<0.001 |
|  | B | Dose main effect | F_2,14_=34.583, p<0.001 |
|  |  | Time main effect | F_4,28_=35.858, p<0.001 |
|  |  | Interaction | F_8,56_=16.024, p<0.001 |
|  | C | Dose main effect | F_2,14_=124.432, p<0.001 |
|  |  | Time main effect | F_4,28_=44.842, p<0.001 |
|  |  | Interaction | F_8,56_=21.914, p<0.001 |
|  | D | Genotype main effect | F_2,21_=0.0202, p=0.980 |
|  |  | Time main effect | F_4,84_=133.661, p<0.001 |
|  |  | Interaction | F_8,84_=1.377, p=0.219 |
| Hypothermia | E | Dose main effect | F_2,12_=8.849, p=0.004 |
|  |  | Time main effect | F_4,24_=79.945, p<0.001 |
|  |  | Interaction | F_8,48_=6.239, p<0.001 |
|  | F | Dose main effect | F_2,14_=19.934, p<0.001 |
|  |  | Time main effect | F_4,28_=43.264, p<0.001 |
|  |  | Interaction | F_8,56_=11.087, p<0.001 |
|  | G | Dose main effect | F_2,14_=38.417, p<0.001 |
|  |  | Time main effect | F_4,28_=54.676, p<0.001 |
|  |  | Interaction | F_8,56_=19.858, p<0.001 |
|  | H | Genotype main effect | F_2,21_=0.342, p=0.714 |
|  |  | Time main effect | F_4,82_=122.586, p<0.001 |
|  |  | Interaction | F_8,82_=0.610, p=0.767 |
| Catalepsy | I | Dose main effect | F_2,12_=13.454, p<0.001 |
|  |  | Time main effect | F_4,24_=7.586, p<0.001 |
|  |  | Interaction | F_8,48_=6.774, p<0.001 |
|  | J | Dose main effect | F_2,14_=5.574, p=0.017 |
|  |  | Time main effect | F_4,28_=12.713, p<0.001 |
|  |  | Interaction | F_8,56_=1.890, p=0.080 |
|  | K | Dose main effect | F_2,14_=17.271, p<0.001 |
|  |  | Time main effect | F_4,28_=14.344, p<0.001 |
|  |  | Interaction | F_8,56_=5.023, p<0.001 |
|  | L | Genotype main effect | F_2,21_=0.150, p=0.862 |
|  |  | Time main effect | F_4,83_=23.873, p<0.001 |
|  |  | Interaction | F_8,83_=0.505, p=0.849 |
| Impaired rotarod performance | M | Dose main effect | F_2,12_=13.017, p<0.001 |
|  |  | Time main effect | F_4,24_=7.525, p<0.001 |
|  |  | Interaction | F_8,48_=8.636, p<0.001 |
|  | N | Dose main effect | F_2,14_=23.934, p<0.001 |
|  |  | Time main effect | F_4,28_=7.131, p<0.001 |
|  |  | Interaction | F_8,56_=4.753, p<0.001 |
|  | O | Dose main effect | F_2,14_=9.179, p=0.003 |
|  |  | Time main effect | F_4,28_=21.009, p<0.001 |
|  |  | Interaction | F_8,56_=9.609, p<0.001 |
|  | P | Genotype main effect | F_2,21_=0.351 p=0.708 |
|  |  | Time main effect | F_4,84_=81.004, p<0.001 |
|  |  | Interaction | F_8,84_=1.125, p=0.355 |

**Supplementary Table 2:** Statistical results of oxycodone-induced analgesia and hypothermia in wildtype littermates (CB1-flox) and Vgat-CB1-KO mice as shown in Figure 5.

| Measurement | Figure 5 | Main effects | Statistical result |
| --- | --- | --- | --- |
| Analgesia | A | Dose main effect | F_2,12_=4.957, p=0.027 |
|  |  | Time main effect | F_4,24_=7.654, p<0.001 |
|  |  | Interaction | F_8,48_=6.792, p<0.001 |
|  | B | Dose main effect | F_2,14_=11.731, p=0.001 |
|  |  | Time main effect | F_4,28_=20.733, p<0.001 |
|  |  | Interaction | F_8,56_=16.795, p<0.001 |
|  | C | Genotype main effect | F_1,13_=0.558, p=0.468 |
|  |  | Time main effect | F_4,52_=26.738, p<0.001 |
|  |  | Interaction | F_4,52_=0.132, p=0.970 |
| Hypothermia | D | Dose main effect | F_2,12_=9.958, p=0.003 |
|  |  | Time main effect | F_4,24_=15.814, p<0.001 |
|  |  | Interaction | F_8,48_=7.864, p<0.001 |
|  | E | Dose main effect | F_2,14_=9.729, p=0.002 |
|  |  | Time main effect | F_4,28_=8.442, p<0.001 |
|  |  | Interaction | F_8,56_=5.885, p<0.001 |
|  | F | Genotype main effect | F_1,13_=0.106, p=0.750 |
|  |  | Time main effect | F_4,52_=49.369, p<0.001 |
|  |  | Interaction | F_4,52_=2.452, p=0.057 |

**Supplementary Figure 4:**

**
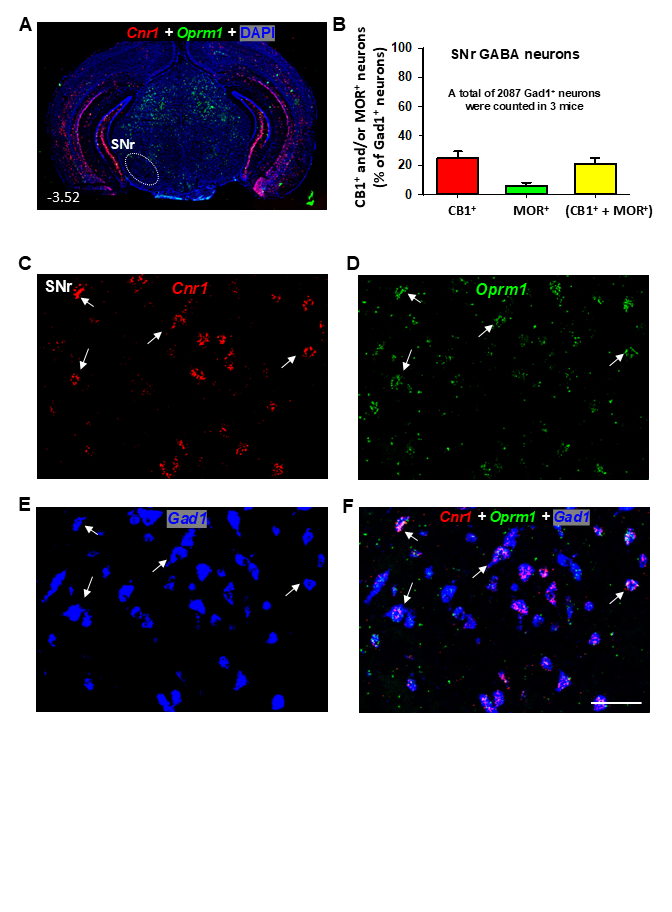
**

**Supplementary Figure 4:** GABAergic CB1R and MOR expression and colocalization in the substantia nigra pars reticulata (SNr) of MOR-flox mice. **A**: Overlaid images (4×) of *Cnr1* and *Oprm1* mRNA in a brain slice at Bregma level of -3.52 mm, illustrating low-density CB1 and MOR expression in the SNr. **B**: Quantitative cell counting data reveal that ~20% of *Gad1^+^* GABA neurons co-express CB1R and MOR, ~25% express CB1R alone, and <5% express MOR alone. The number shown in the bar figure represents the total number of *Gad1*^+^ GABAergic neurons analyzed from the SNr, from which percentages of CB1R^+^ and/or MOR^+^ neurons were calculated. **C**-**F**: Representative high-magnification (40×) images, illustrating a small subset of *Gad1^+^* GABA neurons co-express CB1R and MOR in the SNr. Scale bar: 50 μm.

**Supplementary Figure 5:**

**
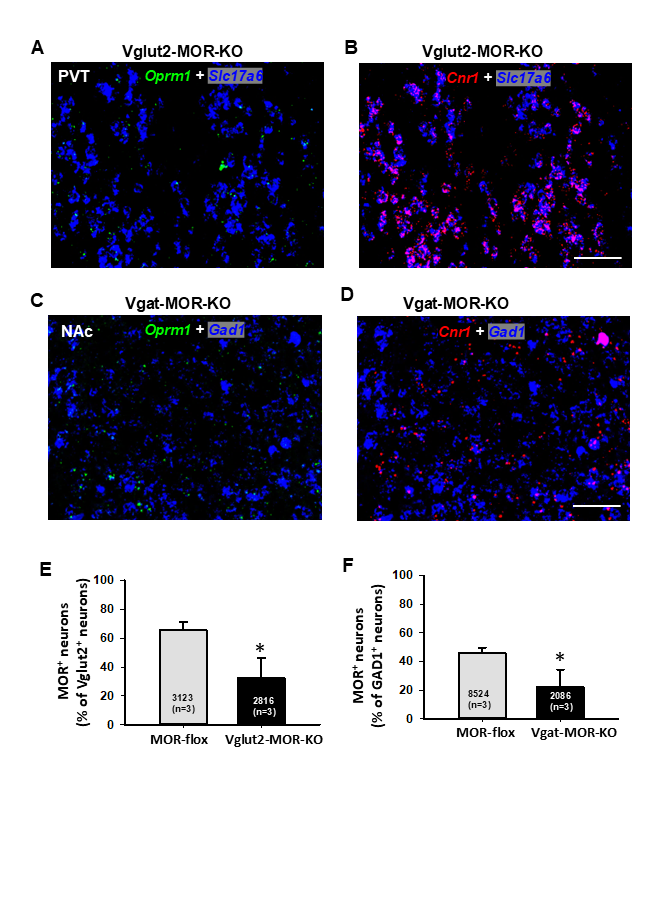
**

**Supplementary Figure 5**: Verification of MOR signal specificity in RNAscope ISH assay. **A/B**: Representative mRNA staining of *Oprm1* (**A**, green), *Cnr1* (**B**, red), and *Slc17a6* (**A, B**, blue) in the same field of the PVT, illustrating that *Oprm1* is undetectable in most *Slc17a6^+^* glutamate neurons (**A**) in Vglut2-MOR-KO mice. In contrast, a high density of *Oprm1* is detected in ~60% of *Slc17a6^+^* glutamate neurons in MOR-flox and CB1-flox control mice (Fig. 1F, G, H). Notably, deletion of MOR from *Slc17a6^+^* glutamate neurons did not alter CB1R expression in these neurons (**B**). **C**/**D**: Representative *Oprm1* and *Cnr1* mRNA staining in NAc GABA neurons of Vgat*-*MOR-KO mice, showing that *Oprm1* is undetectable in most of *Gad1^+^* GABA neurons in the NAc (**C**). However, deletion of MOR from GABA neurons did not alter *Cnr1* expression in Vgat*-*MOR-KO mice (**D**). **E**: Quantitative cell counting results show a significant reduction in the percentage (%) of *Slc17a6^+^* glutamate neurons expressing MOR, from ~60% in MOR-flox mice to ~30% in Vglut2-MOR-KO mice. **F**: Quantitative cell counting results also show a significant reduction in the percentage (%) of *Gad1*^+^ GABA neurons expressing MOR, from ~45% in MOR-flox and CB1-flox mice to ~20% in Vgat-MOR-KO mice. Scale bar = 50 μm. The numbers shown in the bar figure represents the total numbers of *Vglut2*^+^ glutamatergic neurons (**E**) or *Gad1^+^* GABAergic neurons (**F**) counted cross 3 mice in each strain.
